# snOPY: a small nucleolar RNA orthological gene database

**DOI:** 10.1186/1756-0500-6-426

**Published:** 2013-10-23

**Authors:** Maki Yoshihama, Akihiro Nakao, Naoya Kenmochi

**Affiliations:** 1 Frontier Science Research Center, University of Miyazaki, 5200 Kihara, Kiyotake, Miyazaki 889-1692, Japan; 2 Hymena & Co., 1-21-3 Ebisu, Shibuya, 4-1-10 Kounan, Minato, Tokyo 108-0075, Japan

**Keywords:** snoRNA, RNA modification, Intron

## Abstract

**Background:**

Small nucleolar RNAs (snoRNAs) are a class of non-coding RNAs that guide the modification of specific nucleotides in ribosomal RNAs (rRNAs) and small nuclear RNAs (snRNAs). Although most non-coding RNAs undergo post-transcriptional modifications prior to maturation, the functional significance of these modifications remains unknown. Here, we introduce the snoRNA orthological gene database (snOPY) as a tool for studying RNA modifications.

**Findings:**

snOPY provides comprehensive information about snoRNAs, snoRNA gene loci, and target RNAs. It also contains data for orthologues from various species, which enables users to analyze the evolution of snoRNA genes. In total, 13,770 snoRNA genes, 10,345 snoRNA gene loci, and 133 target RNAs have been registered. Users can search and access the data efficiently using a simple web interface with a series of internal links. snOPY is freely available on the web at http://snoopy.med.miyazaki-u.ac.jp.

**Conclusions:**

snOPY is the database that provides information about the small nucleolar RNAs and their orthologues. It will help users to study RNA modifications and snoRNA gene evolution.

## Findings

### Background

Large-scale sequencing and transcriptome analyses have revealed that most of the genome is transcribed and that there are a large number of non-protein-coding transcripts present in the cell [1]. Functional non-coding RNAs (ncRNAs) include micro RNAs (miRNAs), short interfering RNAs (siRNAs), and Piwi-interacting RNAs (piRNAs), which play important roles in biological processes such as gene expression, gene silencing, and RNA processing [2]. In addition, there are many classical essential ncRNAs, including ribosomal RNAs (rRNAs), small nuclear RNAs (snRNAs), and tRNAs. Some of these RNAs are known to undergo post-transcriptional modifications [3-5]. Experimental results have shown that deficiencies in RNA-modifying enzymes lead to embryonic death in mice, and the loss of rRNA modification leads to developmental defects in zebrafish, which signifies the importance of RNA modifications for the proper functioning of ncRNAs [6,7]. Although many modification sites have been identified [8], the functions of these modifications remain unknown.

Small nucleolar RNAs (snoRNAs) play key roles in the RNA modification process. These RNAs function as guide RNAs for the site-specific modification of target RNAs such as rRNAs and snRNAs [9]. Over the last decade, a large number of snoRNAs have been identified experimentally or computationally in various species [10,11]. These RNAs are encoded by three types of genomic loci, i.e., intronic gene loci, polycistronic gene loci (clusters), and monocistronic gene loci (independent) [9]. The snoRNA genes of different loci must be expressed in different ways but in a coordinated manner. For example, for the maturation of human 28S rRNA, 98 distinct snoRNA genes need to be expressed simultaneously from 65 independent loci. It is still unclear how the expression of these snoRNAs is regulated in a synchronized manner.

We have constructed the snoRNA orthological gene database (snOPY) as a tool for studying RNA modifications and snoRNA gene evolution. This database provides comprehensive information about snoRNAs, snoRNA gene loci, and target RNAs. In addition, it includes manually curated orthologous gene data for each gene. This unique database enables users to analyze not only snoRNAs but also their targets and gene organization in various species.

### Database content

snOPY provides three main types of information: snoRNA, snoRNA gene locus, and target RNA (Table 1). As of October 2013, it contains 13,770, 10,345, and 133 records of snoRNAs, snoRNA gene loci, and target RNAs, respectively.

**Table 1:** snOPY statistics

**Classification**	**No.**
Records	
Species	34
snoRNA gene	13,770
Gene locus	10,345
Target RNA	133
Box type	
C/D	4,795
H/ACA	7,913
H/ACA, C/D	2
Unclassified	1,060
Gene locus	
Intronic	2,539
Polycistronic	473
Monocistronic	7,333
Target RNA	
Ribosomal RNA (rRNA)	101
Small nuclear RNA (snRNA)	32

Numbers include both curated and noncurated data. As of October 2013, the number of curated snoRNA gene entries is 2,024.

#### ***snoRNA***

**Figure 1:**
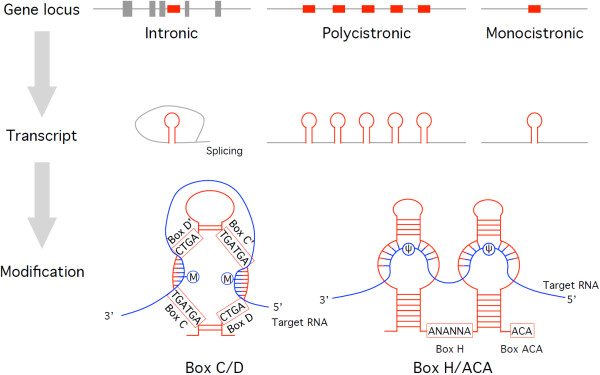
**Secondary structure of snoRNAs and genomic loci.** Three types of snoRNA gene loci (top), intermediate transcripts (middle), and mature box C/D and box H/ACA snoRNAs associated with target RNAs (bottom) are shown. Circles indicate modification sites for methylation (m) and pseudouridylation (Ψ). snoRNAs, snoRNA gene loci, and target RNAs are shown in red, gray, and blue, respectively.

**Figure 2:**
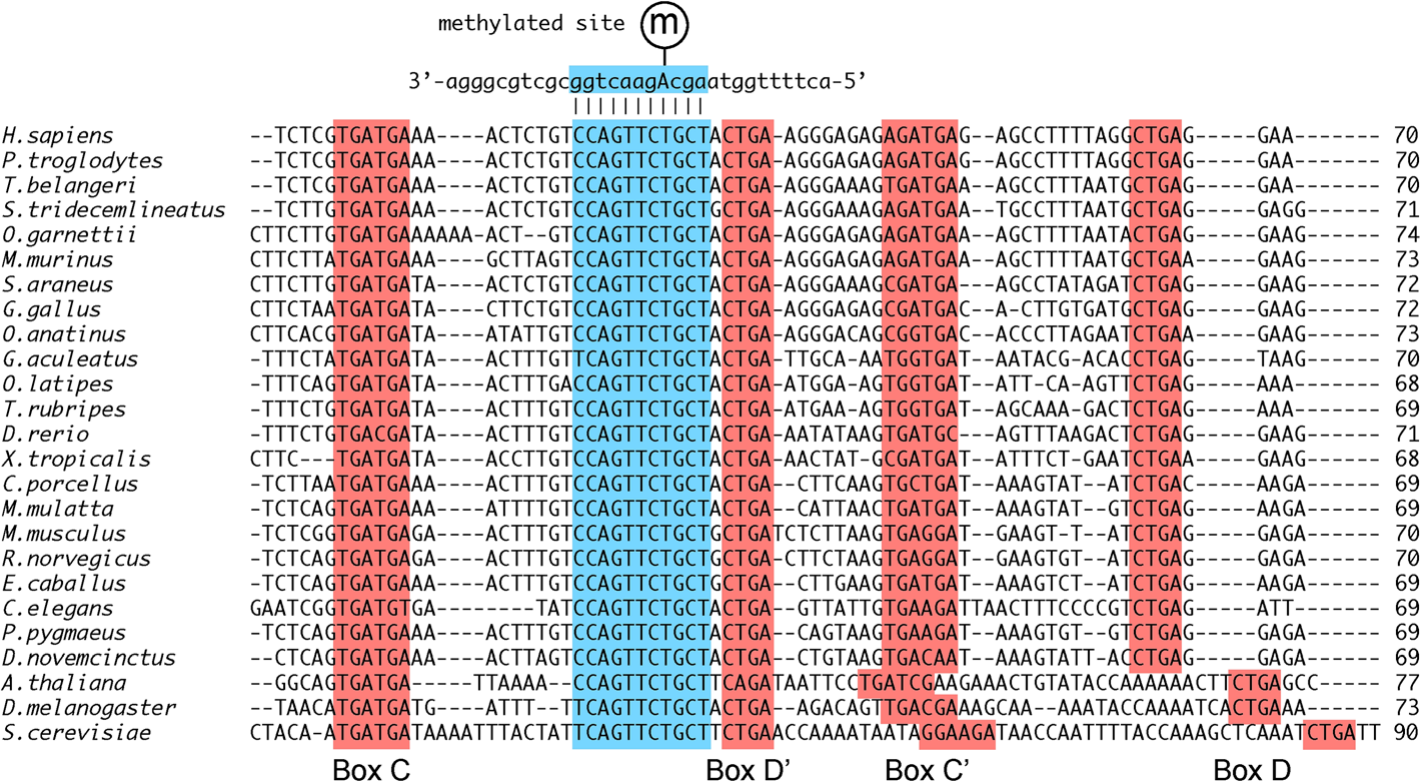
**A multiple sequence alignment of snoRNAs (SNORD38) from 25 species.** Part of the target RNA sequence (*H. sapiens*) and modification site are also included. Box motifs and complementary sequences are highlighted in red and blue, respectively. The multiple alignment was generated by ClustalW [17].

The major function of snoRNAs is to guide the modification of rRNAs or snRNAs via antisense RNA:RNA interactions with their target RNAs (Figure 1). snoRNAs are divided into two major classes based on highly conserved motifs, i.e., the C/D and H/ACA boxes [9]. The C/D box snoRNAs contain two sequence motifs (C box: TGATGA; D box: CTGA) and direct the 2′-*O*-methylation of their target RNAs. In these snoRNAs, a region upstream of the D or D’ box is complementary to the target RNA, and the modification occurs 5 nt upstream of these boxes (Figures 1 and 2) [12]. The H/ACA box snoRNAs also contain two sequence motifs (H box: ANANNA; ACA: ACA box) and guide the pseudouridylation (conversion of uridine to pseudouridine) of the target RNA. The modification site is located at the pseudouridylation pocket, which is formed by an RNA:RNA antisense interaction between complementary sequences of the snoRNA and target RNA (Figure 1) [13]. The snoRNA data were collected from public databases according to the sequence annotation and manually curated.

#### ***Gene locus***

There are three types of snoRNA gene loci: intronic, polycistronic, and monocistronic [9,14]. In intronic loci, the snoRNA gene is located within the intron of protein-coding or non-protein-coding genes (host gene) and transcribed simultaneously with its host gene under the control of the host gene promoter. The maturation of snoRNA transcripts is achieved via the splicing and subsequent processing of the host gene. In the animal kingdom, most snoRNA genes are expressed from introns [14]. The polycistronic loci contain multiple snoRNA genes that are organized into a cluster and transcribed from a single promoter, whereas the monocistronic loci contain a single snoRNA gene that is expressed from its own promoter. In plants and yeast, most of the snoRNA genes exhibit either polycistronic or monocistronic expression [15,16].

#### ***Target RNA***

rRNAs and snRNAs are the major targets of snoRNAs. In general, the number of modified nucleotides depends on the length of the target RNA. For example, human 28S rRNA and U2 snRNA contain 119 and 13 modification sites, respectively. However, there are many orphan snoRNAs whose targets remain to be determined.

#### ***Orthologue***

snOPY also contains information about snoRNA orthologues. The identification of the orthologues using common homology search techniques such as BLAST is difficult because the sequence conservation between snoRNAs from different species is very low (Figure 2). Although there are some short conserved motifs, BLAST often fails to identify the correct counterparts. Therefore, we focused on the sequence conservation between the target RNAs such as rRNAs rather than the snoRNA sequences themselves to identify the orthologues. We performed sequence alignment of the target RNAs from different species using ClustalW [17], then mapped the modification sites on that alignment. If the modified nucleotide is aligned at the same position, we assumed the snoRNA that guides this modification as an orthologue.

### Utility and discussion

**Figure 3:**
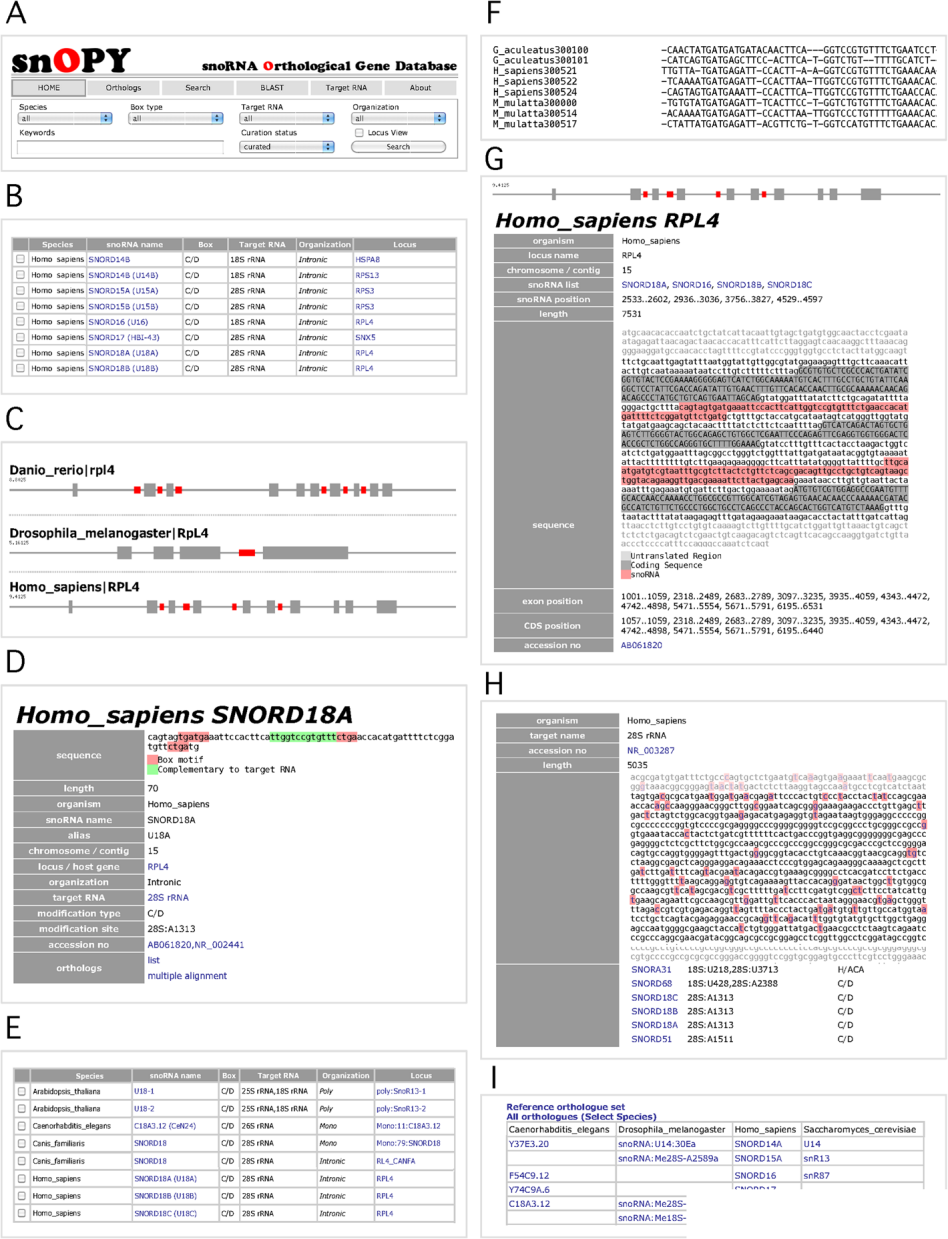
**Representative snapshots of snOPY pages. A**, search form; **B**, search results selected with “*Homo sapiens*”; **C**, results retrieved from “Locus View” using “RPL4” as a keyword; **D**, individual snoRNA entry page for *H. sapiens SNORD18A*, with box motifs and complementary sequences highlighted in red and green, respectively; **E**, orthologues retrieved using “list” in the human *SNORD18A* page; **F**, multiple sequence alignment for *SNORD18A*; **G**, snoRNA gene locus of the human *RPL4* gene for *SNORD18A*; **H**, target RNA and modification sites for human 28S rRNA; **I**, an orthologue table for four representative species. With the exception of **A** and **C**, only a part of each page is shown in the snapshot.

snOPY provides several search parameters, including species, box motif, target RNA, gene organization, curation status, and keywords. Users can also perform a BLAST search for the gene sequences, gene loci, and target RNAs (Figure 3A, 3B). In addition, search results are visualized using “Locus View”, which enables users to compare the snoRNA locus directly between various species (Figure 3C).

Each snoRNA entry page provides basic information about the locus, including the snoRNA gene sequence, type of box motif, and genomic position (Figure 3D). Information relating to the gene locus and target RNA is also provided, and these items are linked to more detailed descriptions (Figure 3E). Users can retrieve orthologues and perform multiple sequence alignments via this page (Figure 3F). The locus entry pages show schematics of the locus structure and sequence, as well as other information about the locus (Figure 3G). The target RNA entry pages show complete RNA sequences and modification sites (Figure 3H). When available, the snoRNAs involved in these modifications are also shown, with links to the individual snoRNA entry page. Users can access a list of all target RNAs via the “Target RNA” link at the top of each page (Figure 3A).

The orthologues table page shows the orthologous relationships between snoRNA genes from various species (Figure 3I). The default setting includes four selected species, *Homo sapiens*, *Caenorhabditis elegans*, *Drosophila melanogaster*, and *Saccharomyces cerevisiae*, which are well studied and widely referenced species. Users can select any species for comparison and readily access the reference data from the default setting.

At present, there exist several other databases for snoRNAs, including snoRNA-LBME-db [18], Yeast snoRNA Database [16], Plant snoRNA Database [19], and the sno/scaRNAbase [20]. These databases provide very useful information about the snoRNAs from particular organisms. However, users are unable to compare the snoRNAs from various species. On the other hand, snOPY provides data from a wide variety of species, which enables users to perform comparative analysis very efficiently.

## Availability and requirements

snOPY is freely available on the web at http://snoopy.med.miyazaki-u.ac.jp.

## Competing interests

The authors declare that they have no competing interests.

## Authors’ contributions

MY designed and implemented the database. AN designed and developed the web server. NK designed and developed the database and wrote the manuscript. All authors read and approved the final manuscript.
